# Study on the Polarization of Random Lasers from Dye-Doped Nematic Liquid Crystals

**DOI:** 10.1186/s11671-016-1778-x

**Published:** 2017-01-11

**Authors:** Lihua Ye, Chong Zhao, Yangyang Feng, Bing Gu, Yiping Cui, Yanqing Lu

**Affiliations:** 1 Advanced Photonics Center, Southeast University, Nanjing, Jiangsu 210096 China; 2 College of Engineering, Applied Sciences and National Laboratory of Solid State Microstructures, Nanjing University, Nanjing, Jiangsu 210093 China

**Keywords:** Random lasers, Liquid crystals, Polarization

## Abstract

Random lasers from dye-doped nematic liquid crystal (DDNLC) cells with different rubbing methods were observed due to different random ring cavities that were formed. Through constructing cells with different rubbing methods on the forward and backward surfaces of light-emitting sides, we can get two random laser beams with different polarization directions from one DDNLC cell at the same time, and the polarization direction is along the rubbing direction of the light-emitting sides. Additionally, the influence of external electric field on the polarization degree of random lasers was also studied.

## Background

Random lasers get gain from multiple scattering. Compared with conventional lasers, the resonant cavity in random lasers is built on recurrent multiple scattering instead of two mirrors with high reflectivity. The fluorescence photons can be repeatedly multi-scattered in random directions as they propagate in an active medium where the scattering particles or domains are distributed disorderly. The recurrent multiple scattering can form incoherent feedback or coherent feedback, when the scattering photons propagate along a close circuit [1]. Random laser can be obtained light pumped or electrically pumped [2]. With advantages of small size, cheapness, flexible shape, and some others [3], random lasers can be widely used in temperature sensing [4], document encoding, material marking, high-density optical data storage [5], tumor diagnosis [6, 7], liquid crystal display [8], integrated optics [3], liquid flow monitoring, and other areas [9].

In recent years, random lasers have been developed using liquid crystals (LCs) as a scattering material. As a typical anisotropic material, the LC has a lot of optoelectronic applications because of its unique optoelectronic properties. The LCs can be divided into nematic phase, cholesteric phase, and smectic phase. The choosing of different LC materials can achieve different emission characteristics of LC random laser [10–13]. Such random lasers possess peculiar merits of flexible controllability or tunability in their lasing characteristics (e.g., energy threshold or lasing wavelength) by thermal [14, 15], electric [16, 17], and optical [18] approaches. This is because the orientation of LCs with large anisotropies and its macroscopic physical properties, e.g., the refractive index and dielectric tensor, can be easily modified externally. Additionally, it is known that sample parameters affect the optical properties of the LC cells in the field of liquid crystal display [19] and these properties such as polarization can be controlled through changing the parameters. Nematic liquid crystal (NLC) is a kind of anisotropic material, which has the properties of birefringence. The change of temperature will affect the birefringence and light scattering of NLC. NLC molecules are aligned in parallel along the long axis of the molecule, and the liquid crystal molecules are in the shape of a rod. Therefore, the nematic liquid crystal molecules are easily affected by the applied electric field.

The polarization state of emission spectrum is an important characteristic for the random laser. In 2004, Wu et al. investigated RLs in a two-dimensional rod array. Their results showed that due to the strong scattering of the random lasers, the transverse magnetic-polarized lasing component had a lower threshold than the transverse electric (TE)-polarized component [20]. At the same year, Gottardo et al. used extremely anisotropic scattering from small droplets of liquid crystals to create and manipulate polarized random laser emission [21]. In 2012, it was found that random lasers in organic dye solutions can be linearly polarized using the anisotropic adsorption of the dye molecules [22]. In [23], random laser emitted from DDNLCs was investigated, and any arbitrary linear polarization of RLs can be obtained by rotating the nematic liquid crystal sample. These researches have blazed a way in polarization study in different structure systems for us.

In this paper, the polarization of random lasers from DDNLCs was studied through changing the rubbing methods of the LC cells and the external electric field. By using different rubbing methods on the light-emitting sides, random laser with different polarization directions can be obtained from both forward and backward surfaces of one cell at the same time. With increasing the electric field intensity, the polarization degree of random lasers was reduced clearly.

## Methods

**Fig. 1:**
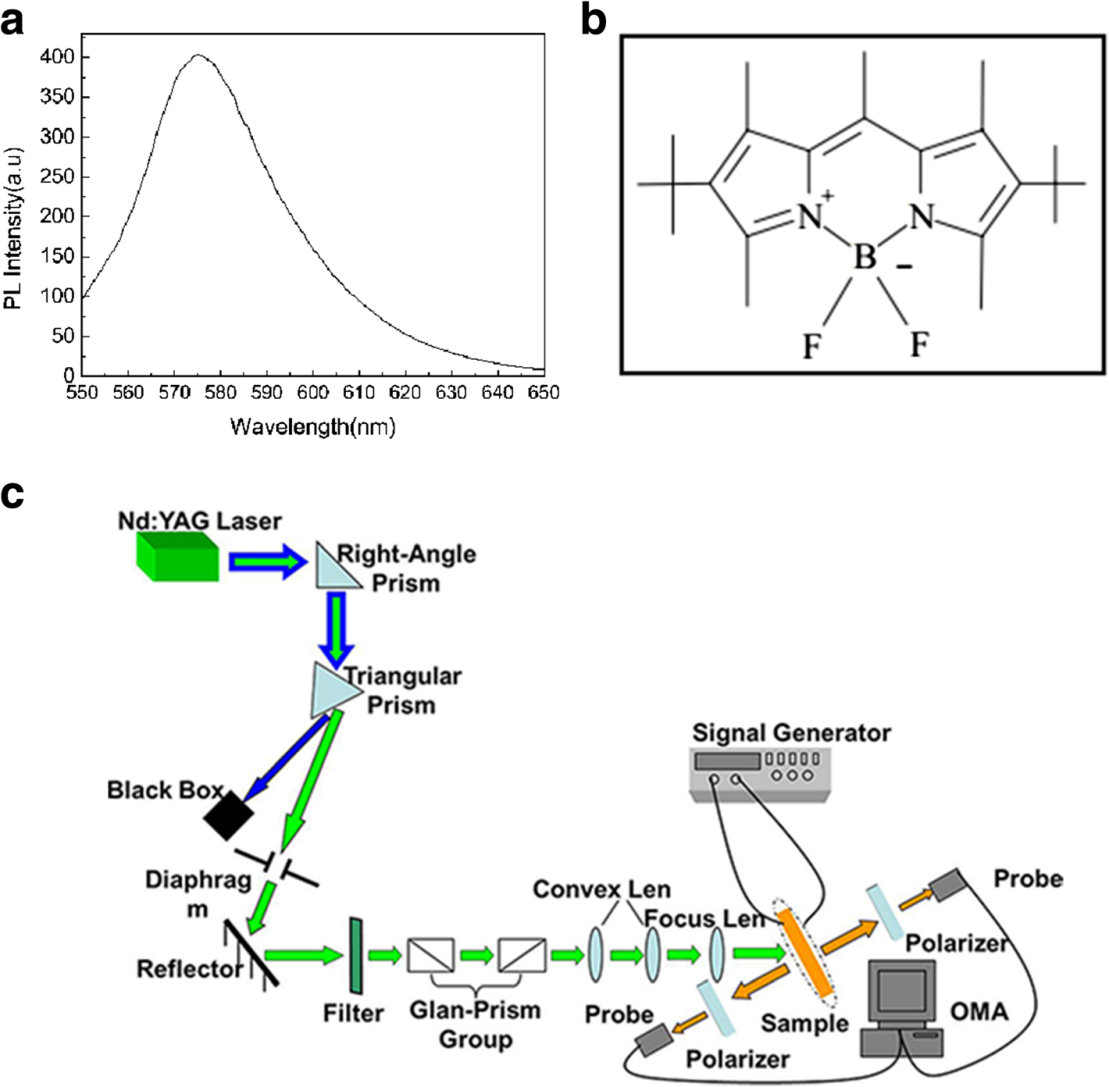
**a** The fluorescence spectrum of laser dye PM597. **b** The molecular structure of the laser dye PM597. **c** The sketch of the experimental setup

Firstly, the empty cells were constituted by two glass substrates separated by Mylar slices which decide the cell gap. The thickness of Mylar slices was 100 μm. The glass substrates were covered with rubbed polyimide alignment layers to induce a homogeneous alignment of the LC molecules at the interface. The aligning direction of LCs was decided by rubbed polyimide alignment layers. Then, the mixtures containing liquid crystals E7 (*n*_*o*_ = 1.521 and *n*_*e*_ = 1.746, 99.7 wt.%) and dye PM597 (0.3 wt.%) were injected into the empty cells by the capillary effect. The fluorescence spectrum of PM597 is shown in Fig. 1a. The molecular structure of the laser dye PM597 is shown in Fig. 1b. The dye PM597 has a molecular weight of 374.32, which has a high quantum efficiency. Four sides of the cells were sealed with AB adhesive. Through this method, DDNLC cells with different rubbing methods were made, including “no rubbing (NR),” “one-side rubbing (OSR),” “two-side rubbing in same direction (TSRS),” and “two-side rubbing in vertical direction (TSRV).”

Figure 1b shows the sketch of the experimental setup. The sample was pumped by a frequency doubled Nd:YAG laser system (PowerLite Precision II 8010) with 532-nm wavelength, 10-Hz repetition rate, and 8-ns pulse width. The strength of the pump laser can be changed by adjusting the polarization direction of the Glan prism group. The diameter of laser pump spot was about 20 μm. Random lasers were emitted from both the forward and backward sample surfaces. The emitting signal is collected by optical multichannel analyzer (OMA) with spectral resolution of 0.1 nm.

## Results and Discussion

**Fig. 2:**
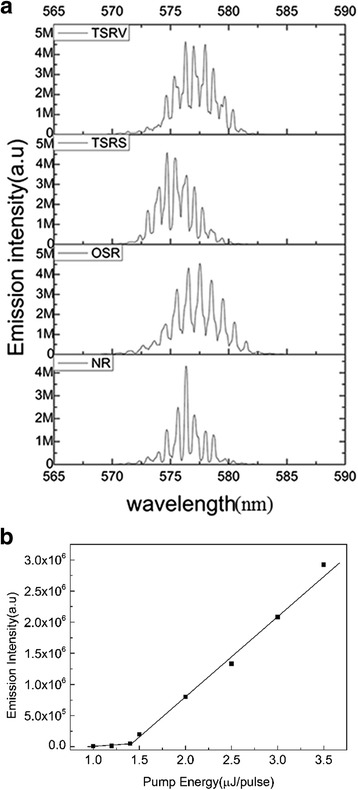
**a** Emission spectrums of the random laser from samples with different rubbing methods (TSRV, TSRS, OSR, NR) when the pump energy is 12 μJ/pulse. **b** The relationship of light emission intensity and the pump energy

Figure 2a shows the emission spectrum of random laser from cells with different rubbing methods. The optical feedback of the random laser is provided by LC multiple scattering. A closed loop path formed by light multiple scattering is analogous to a random microcavity, and the laser oscillation occurs in the random microcavities, which increase dramatically the photon propagation path length in the sample [24]. When the amplification along the microcavity exceeds the loss, random laser operation can occur in the LC cells. As the pump energy exceeds the threshold energy, well-distinguished sharp spikes with linewidth less than 1 nm appear around 576 nm, as shown in our early work [25], indicating that the random lasing occurs. The laser emission from these resonators results in a small amount of sharp peaks in the emission spectrum. The appearance of sharp spikes implies the interference of waves which is usually resulted from intensified scattering in random lasing media. The threshold of random laser is about 1.4 μJ/pulse for TSRS cells with 100-um thickness, as shown in Fig. 2b. In the various cells, there exists red-shift or blue-shift without obvious regularity. This is because there are multiple closed loop paths in the system, which serves as ring cavities for light. The laser wavelengths are determined by the microcavity resonances depending on relevant and equivalent cavity lengths. Different closed loop paths correspond to different cavity lengths, which decide the frequency of the emission peaks, and the modes are different for various cells. So red- and blue-shifts are observed in Fig. 2a. Along different light paths, the probability and distance of a photon scattering back to its starting point are different [26].

In previous reports [27–29], ZnO nanostructures exhibit a whispering gallery mode (WGM) type of resonance in which the hexagon-shaped nanostructure can support the lasing modes. The photons are confined by the total internal reflection at the ZnO-air boundary, where lasing occurs in WGMs with a high Q factor. Different with the WGM lasing which the emission peaks can be attributed to the confined resonant modes inside the hexagon-shaped structure, which is nicely reproduced in Fig. 4c of [28], the optical feedback of the random lasing is provided by the multiple scattering of LC molecules. On the other hand, the threshold of random lasing depends on the excitation area [28], actually because the numbers of LCs are different under various excitation areas, which have an effect on scattering strength and pump efficiency of random laser. The number of LC molecules can also be adjusted by changing the thickness of the samples, which can affect the threshold of random laser. As shown in our previous work [30], the influence of the LC cell gap on random laser energy threshold was studied, and a wedge cell with TSRS rubbing method was made. The results of the experiment show that the scattering strength is different in the distinct thickness of LC cells, which lead to the changes of random laser threshold. However, the pump threshold of WGM is not affected by the change of the excitation area in spite of the increasing number of peaks [28]. Based on the above analysis, the possibility of WGM laser is excluded. It is worth noting that the emission spectrums have almost same frequency spacing. Based on the study of Cao [31], this Fabry-Pérot cavity-like emission in dye-doped nematic liquid crystals can be understood in the way that the random lasers can be regarded as random distributed feedback lasers [32]; the quasimodes are formed mainly by the feedback from weak scattering particles near the system boundary, resulting in quasimodes with almost regular frequency spacing, and similar experimental results have been verified and explained in our previous work [33].

**Fig. 3:**
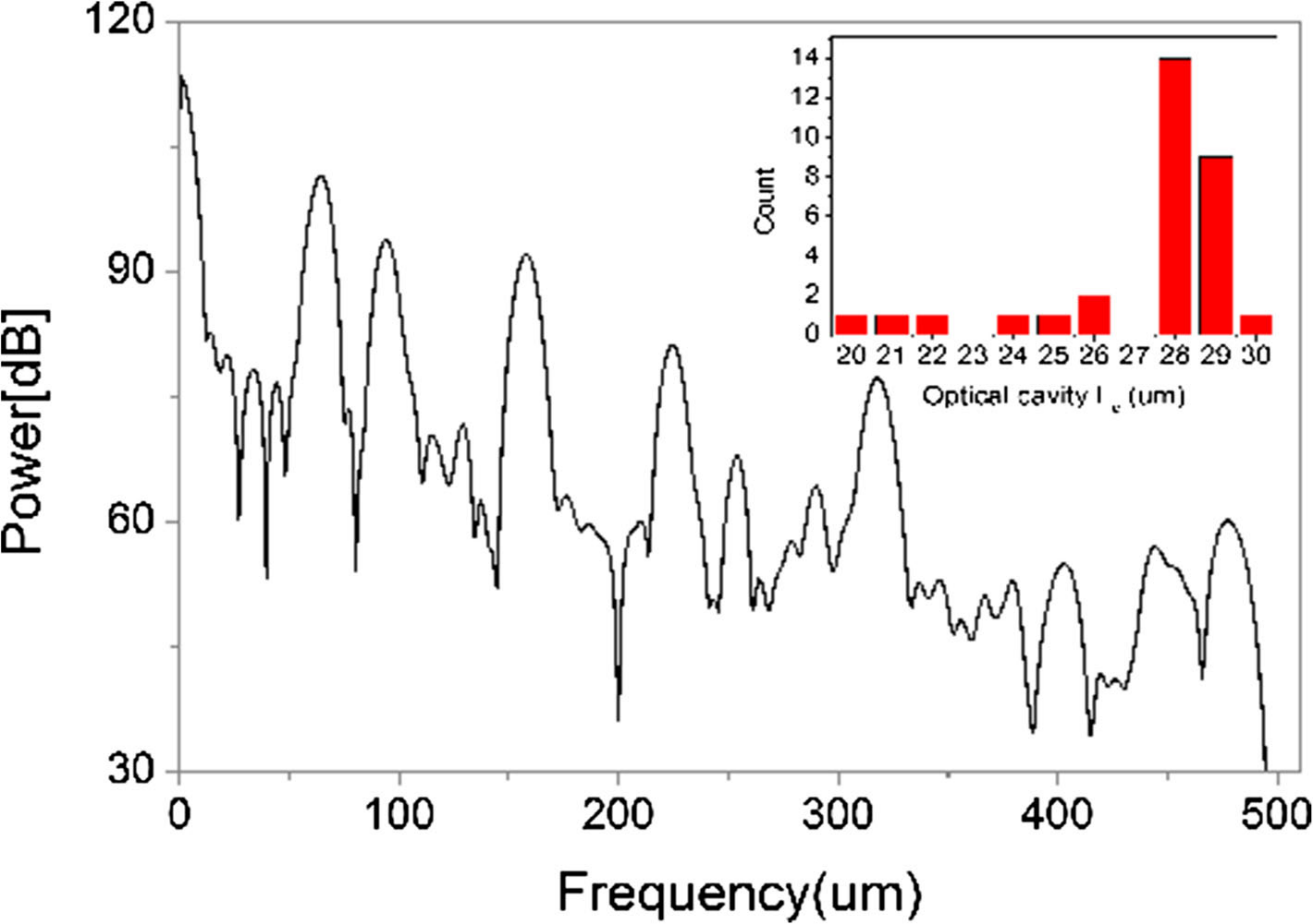
Calculated results of power Fourier transforms of lasing emission spectra. The *inset* shows a histogram for the excited random cavity lengths

In order to derive the excited random ring cavity lengths from the parameters used in the research, the power Fourier transforms (PFT) of the random laser spectra of Fig. 2 are calculated and the results are presented in Fig. 3. As it is well known, the Fourier analysis was performed for random laser spectra represented in *1/λ* scale, so the frequency of obtained Fourier spectra are in micrometer scale [34]. The cavity length *L*_*c*_ is given by the following expression: *L*_*C*_ *= πp*_*m*_ */nm*, where *m* is the order of the Fourier harmonic, *p*_*m*_ is the peak at Fourier’s plot, *L*_*C*_ is the cavity path length, and *n* is the refractive index of the gain medium. The mean optical cavity length and uncertainty determined from PFT analysis are *L*_*c*_ = 27.30 μm and *σ* = 2.79 μm, respectively. Here, *σ* stands for standard deviation. Notice that the average cavity lengths are far smaller than the thickness of cells, which means that the feedback coming from multiple scattering provided by liquid crystals rather than Fabry-Pérot resonator constituted by cell walls forming two reflecting mirrors and similar to that reported in our previous work [35].

**Fig. 4:**
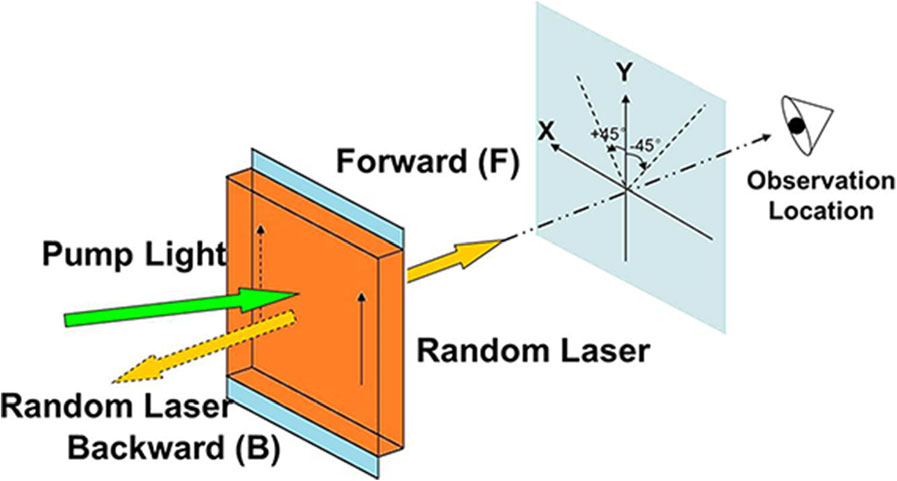
The sketch of the coordinate system and observation location

Then, random lasers with linear polarization from both the forward and backward surface of the DDNLC cells were observed. Figure 4 shows the sketch of coordinate system, observation location, and the setting of the angles, and these conditions were remained unchanged in the experiment for both the forward random laser and backward random laser. The polarization direction of pump laser was fixed along the Y-axis.

**Fig. 5:**
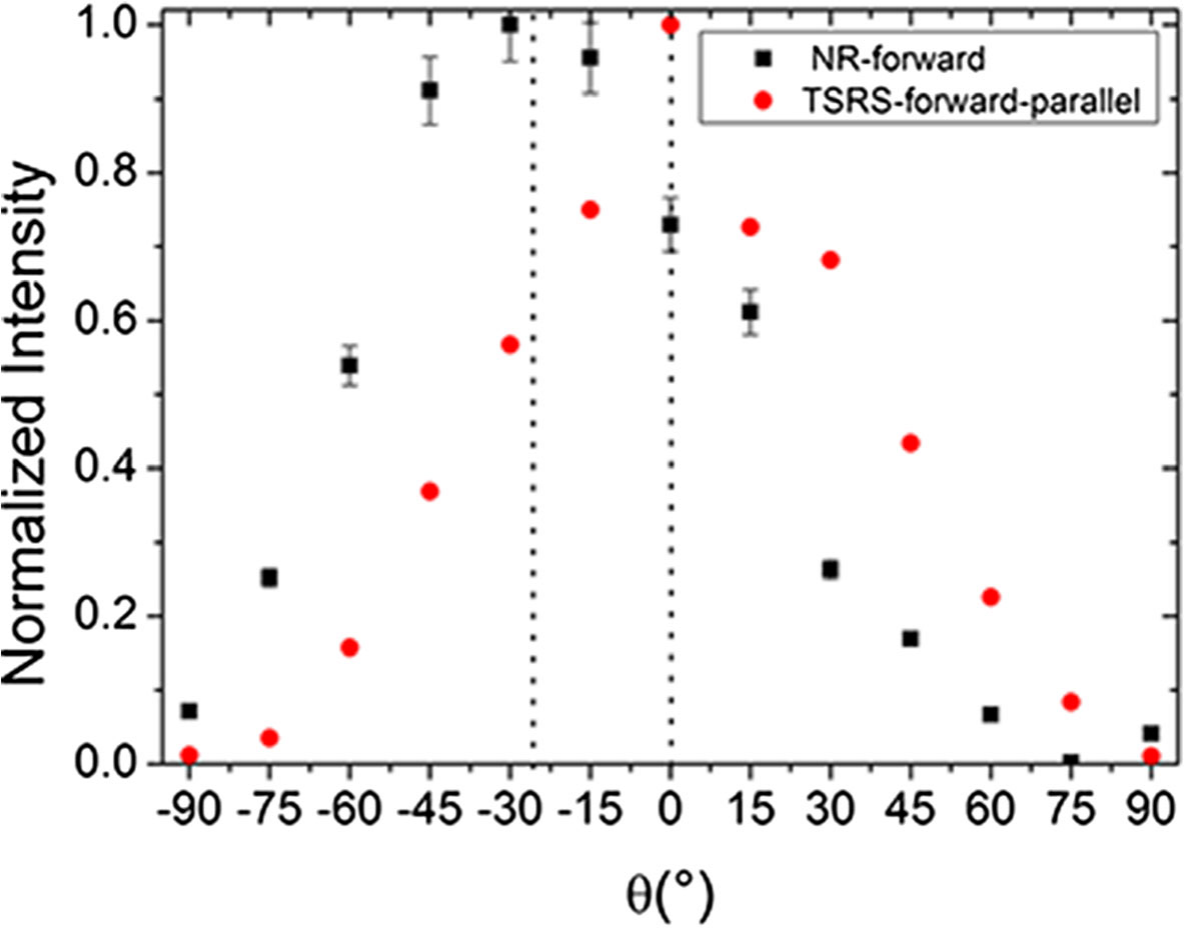
Normalized polarized emission of the forward random laser from NR cells and TSRS cells

It is found that the alignment of liquid crystals influences the polarization of the forward random lasers, as shown in Fig. 5. *θ* is the angle between polarization direction of the pump light (0°) and the polarizer. For NR cells, the liquid crystal molecules are randomly arranged, and the output energy is largest when the *θ* is about 30°. Different with the NR cells, the rubbing methods can control the alignment of liquid crystals, so the polarization direction of the random laser from TSRS cells is consistent with the rubbing direction. When the rubbing direction of TSRS cells is parallel to the polarization direction of the pump light, the emission intensity reaches the largest.

**Fig. 6:**
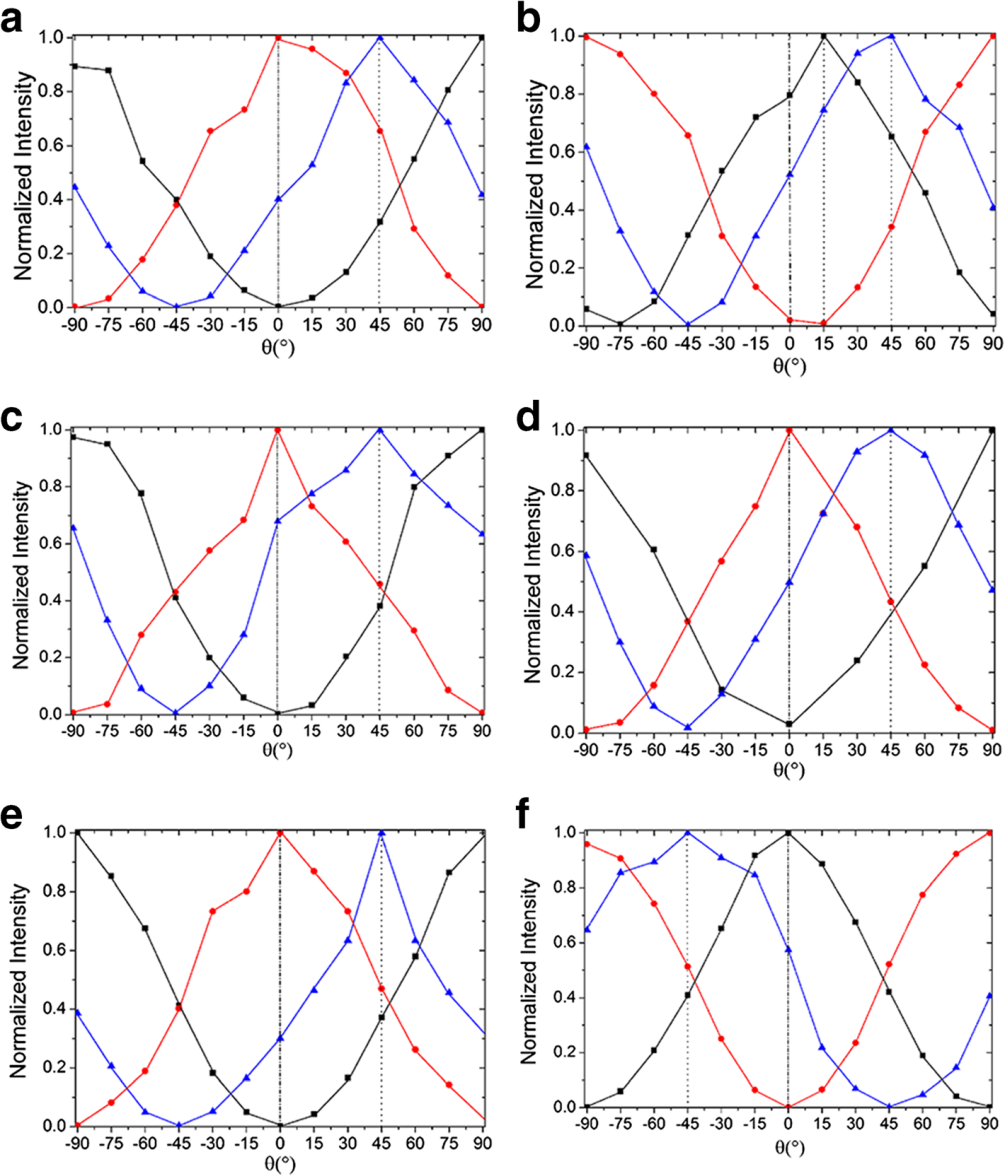
Normalized polarized emission intensity of the forward random laser and backward random laser from OSR cell **a** forward **b** backward, TSRS cell **c** forward **d** backward, and TSRV cell **e** forward **f** backward, when the rubbing directions of the F side are vertical (*square*), parallel (*circle*), and 45°(*triangle*) from Y-axis

Furthermore, in order to verify the influence of different rubbing methods on the polarization of DDNLC random laser operation, both the forward random lasers and backward random lasers were studied, as shown in Fig. 6. For the convenience of description, a side of the cells that emits forward random lasers is called the F side and the other side that emits backward random lasers is called the B side. The fixed position of all the samples is defined according to the F side of the cells, which means the rubbing directions of the F side are vertical, parallel, and 45° from the polarization direction of the pump light (Y-axis). For the various rubbing samples, it can be seen that the polarization direction of the forward random laser is always along the rubbing direction of the F side, as shown in Fig. 6a, c, e. Similarly, in the Fig. 6d, f, the polarization direction of the backward random laser is always along the rubbing direction of the B side except OSR cell, because there is no rubbing behavior in its B side. So, it can be concluded that the polarization direction of random lasers from DDNLC cells is influenced by the rubbing methods and is always along the rubbing direction of the light-emitting side for both forward and backward surfaces of the cells.

This phenomenon can be explained by the anisotropic adsorption of the dye molecules. Random lasers in organic dye solutions can be highly linearly polarized by choosing a highly viscous solvent for the anisotropic adsorption of the dye molecules [36]. There is guest-host effect in dye-doped nematic liquid crystals. According to this effect, rod-shaped dye molecules as “guest” molecules aligned along the direction of the rod-shaped “host” liquid crystal molecules [37, 38]. Dye molecules will tend to absorb light with the polarization direction along the long axis of the dye molecules [39]. When the dye molecules release the energy again, the polarization direction of the emitted light is along the long axis of the dye molecules, which is the direction of the nematic liquid crystal [23]. Due to the rubbing methods controlling the alignment of liquid crystals, the polarization of the emitted light is influenced by the rubbing direction. In the experiment, the anchor force formed from the rubbing behavior is largest for the liquid crystal molecules near the cell surface while is smallest for that in the central of the cell. The inner liquid crystal molecules, which keep away from the cell surface, are not along the nematic director decided by the rubbing direction due to the weak anchor force. However, anchor force will make the alignment of liquid crystal molecules along the orientation direction near the alignment layer, and the light paths can form different loops, as previously mentioned. When the pump light propagates through the cells and produces the random laser, the light with the polarization direction along the rubbing direction will get the largest gain. However, it is hard to get any gain by the light whose polarization direction is not along the rubbing direction. So, the polarization direction of the emitted random laser will gain along the rubbing direction of the cells due to the polarization-dependent optical gain effect. This phenomenon exist in both the forward random laser and backward random laser. This effect can be used to get different random laser beams with different polarization directions from one cell at the same time, which is useful in liquid crystal display and some other fields that polarized light is needed.

**Fig. 7:**
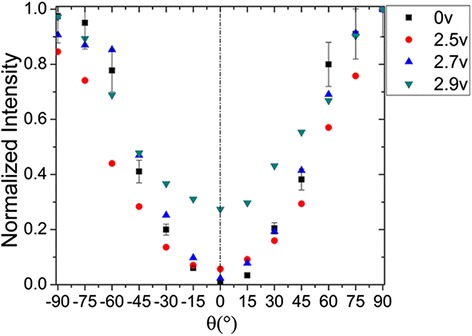
Normalized polarized emission of the forward random laser from TSRS cell changes when the external electric field increases

Electric field can also influence the polarization of random laser from DDNLCs. In our previous work [16], the LC cells with three different rubbing alignments were used to study the electrically controllable random laser from dye-doped liquid crystals, including NR, OSR, and TSRS. And the lowest threshold voltage was obtained in the TSRS cells with various cell gaps, as shown in Table 1, due to the minimum disorder degree of LC molecules. In the experiment, signal generator generated a 1-kHz square wave signal to offer the external alternating voltage, which was applied on the TSRS cells. When the voltage increases, the polarization direction of the random laser will change, as shown in Fig. 7. The same experiment was operated in the literature, while an external electric field with a frequency of 500 Hz is applied to the sample [21]. The transparency of LC cells can be controlled by applying an external electric field, which affects the intensity of polarized light. In one range, the transparency of the LC cells increases with the enhancement of the electric field strength, and this is not related to the frequency of the electric field [21]. As we all know, the polarization degree is calculated by *P = |I*_max_ *− I*_min_ *|/(I*_max_ *+ I*_min_ *). I*_max_ and *I*_min_ are the maximum and minimum intensities of random lasers detected from the detection polarizer. As shown in Fig. 7, when the voltage increases from 0 to 2.9 V, the polarization degree reduces clearly from 0.9932 to 0.5701. The polarization degree of the random laser under different applied voltage was observed in Table 1. Within the acceptable experimental errors, the random laser changes from linear polarization to nearly elliptical polarization. Above the voltage threshold, the inner liquid crystal molecules will realign along the direction of the electric field, thus vertical to the surface of cells, which induce the alignment of dye molecules accordingly. When the anchor force for the inner liquid crystals from the rubbing behavior weakens, the gain gotten by the light with polarization direction along the rubbing direction weakens. But for the liquid crystals near the cell surface, the anchor force formed from the rubbing is still strong enough, so these liquid crystal molecules are fixed in their origin location. So, the difference value of the intensity between the light with polarization direction along the rubbing direction and the light with other polarization direction reduces with the electric field increasing, which will reduce the polarization degree of random lasers.

**Table 1:** The polarization degree of the random laser under different voltage

Voltage (V)	0	2.5	2.7	2.9
Polarization degree	0.9932	0.8927	0.9551	0.5701

## Conclusions

In conclusion, the influence of rubbing methods and external electric field on the polarization of random laser from dye-doped nematic liquid crystals cells is studied in this paper. Random lasers with linear polarization can be obtained from both forward and backward surfaces of the DDNLC cells and the polarization direction is along the rubbing direction of the light-emitting side. Two random laser beams with different polarization directions from one cell can be obtained through constructing cells with different rubbing methods on the forward and backward light-emitting sides at the same time. In addition, increasing external electric field intensity can reduce the polarization degree of random lasers and change the polarization direction of the random lasers. The results reported in this paper can be used in liquid crystal display and some other fields which need light with tunable polarization.
